# Effectiveness of Pelvic Proprioceptive Neuromuscular Facilitation on Balance and Gait Parameters in Children With Spastic Diplegia

**DOI:** 10.7759/cureus.30571

**Published:** 2022-10-22

**Authors:** Vikrant G Salphale, Rakesh K Kovela, Moh'd Irshad Qureshi, Pallavi Harjpal

**Affiliations:** 1 Neuro-Physiotherapy, Ravi Nair Physiotherapy College, Datta Meghe Institute of Medical Sciences, Wardha, IND; 2 Physiotherapy, Nitte Institute of Physiotherapy, Mangalore, IND; 3 Physiotherapy, Ravi Nair Physiotherapy College, Datta Meghe Institute of Medical Sciences, Wardha, IND

**Keywords:** gait parameters, balance, task-oriented training, pelvic proprioceptive neuromuscular facilitation, pelvic congruency, spastic diplegia

## Abstract

Background

Among several variants of Cerebral Palsy, Spastic Diplegic is encountered most commonly in clinical setups. A majority of children with Spastic Diplegia manifest themselves with a disturbance in the geometrical orientation of their pelvis, which imposes an effect on their functional capabilities like walking with independence. This research had an emphasis on the extraction of the efficacy of Pelvic Proprioceptive Neuromuscular Facilitation (PNF) Techniques on Balance and Gait Parameters in children suffering from Spastic Diplegia.

Method

Participants included in the study were between the age groups of 8 to 12 years who were diagnosed with Spastic Diplegia with an independent sitting and walking ability and who are coming in stages I to III according to Gross Motor Function Classification System. Subjects in group A were given Pelvic PNF techniques for 15 minutes on both sides along with Task-Oriented training for 30 minutes, six days a week and continuously for four weeks, while the subjects in group B were given only Task-Oriented activity for the same duration. The pre- and post-treatment assessments of all 40 subjects were gathered using the Paediatric Balance Scale, Palpation Meter device, and Gait Parameters.

Results

The study included 40 participants, which were segregated into two groups of 20 subjects in each group. Group A received Pelvic Proprioceptive Neuromuscular Facilitation with Task-Oriented Training, and group B received only Task-Oriented training activities. The contrast of pre- and post-treatment findings of both the groups revealed that group A reported a significant improvement in their outcomes (P>0.0001).

Conclusion

The present study, which included 40 subjects, has generated evidence regarding the efficacy of Pelvic PNF on Balance and Gait Parameters in children with Spastic Diplegia.

## Introduction

Cerebral Palsy (CP) represents a group of permanent disorders of movement and posture causing activity limitations that are attributed to non-progressive disturbances that occurred in the developing fetal or infant’s brain [1]. Cerebral Palsy has a heterogeneity of risk factors that are responsible for its occurrence as well as in clinical picturization, the extent of severity in the functional limitations, etc. [2]. The causes of Cerebral Palsy are grouped into three segments antenatal, neonatal and postnatal among all these causes of Cerebral Palsy, low birth weight and prematurity are most prevalent [3]. The prevalence of Cerebral Palsy in developing or evolving countries or nations is around two babies per 1000 live births [4]. The prevalence of Cerebral Palsy in India is around 2.95 per 1000 babies [5]. Among several subtypes of Cerebral Palsy, the Spastic Diplegic variant is the most common subtype, and a majority of sufferers with Spastic Diplegia manifest themselves with an asymmetry in their pelvis [6]. Spasticity is an abnormal rise in muscle tone that relies on the velocity of a movement, and it is manifested as a feature of pyramidal tract lesions [7]. Children with Cerebral Palsy are classified or categorized into five heterogenous stages according to the Gross Motor Function Classification System based on their gross motor functioning capabilities or potencies [8]. Other than Gross Motor Function Classification System, some other systems that categorize a child suffering from Cerebral Palsy are the manual ability classification system and the eating and drinking ability classification system [9-10]. Children with Spastic Diplegia often suffer from many functional deficits due to the emergence of numerous musculoskeletal defacements like mal-alignment in their pelvis [11]. It is necessary to have a good synchronization between the pelvis and inferior limbs for a healthy pattern of gait [12]. In the current era, there are numerous strategies of treatment for Cerebral Palsy pharmacological and surgical options, as well as physiotherapy approaches [13]. Physiotherapy approaches that are currently available for the treatment of Cerebral Palsy are Neurodevelopmental therapy (NDT), Proprioceptive Neuromuscular Facilitation (PNF), Constraint-induced movement therapy (CIMT), and many more [14]. Previous research has shown and proven the efficacy of Pelvic PNF in restoring and optimizing control over the trunk in children with Spastic Diplegia [15]. Therefore, the current study had an emphasis on the optimization of balance and gait parameters by normalizing the pelvic geometry.

## Materials and methods

**Figure 1:**
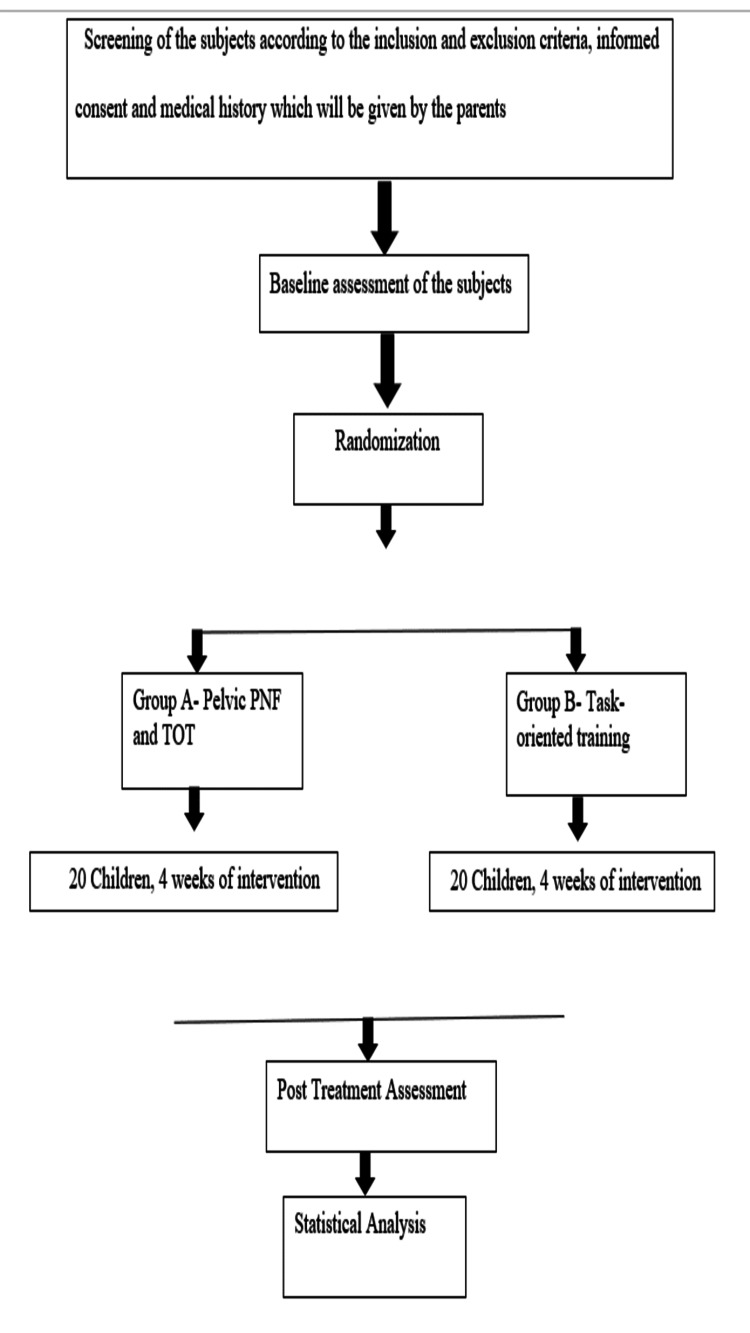
Flow chart of the entire study procedure, which shows the segregation of 40 samples in two groups named A and B in which group A received Pelvic Proprioceptive Neuromuscular Facilitation (PNF) and Task-Oriented Training (TOT), and group B received only Task-Oriented Training (TOT)

After receiving the ethical approval from the Institutional Ethical Committee of Datta Meghe Institute of Medical Sciences (DMIMS) (Ethical permission number: DMIMS [DU]/IEC/2021/249), the study was conducted in the outpatient department (OPD) of Neuro-Physiotherapy, and the participants referred to the OPD of Neuro-Physiotherapy from the Inpatient and outpatient department of Paediatrics at Acharya Vinoba Bhave Rural Hospital, Sawangi, Meghe, Wardha, Maharashtra. Before the commencement of treatment, the subjects were asked to fill out the consent forms given by the investigator, and before giving the consent forms to all the subjects, they were guided about the way of filling out the consent forms. After obtaining the consent forms, the pre-treatment or baseline findings were gathered from all the subjects using the Paediatric Balance Scale, Palpation Meter device, and Gait Parameters. The inclusion criteria of the subjects for the study were the age group between 8-12 years, either gender, independent sitting and walking abilities, levels of Gross Motor Function Classification System ranging from I to III, and Mini Mental Status Examination of more than 22. While on the other hand, the criteria for exclusion of the subjects were children who had undergone any surgical procedure of the spine or lower limbs in the past six months, children who were under botox medication for the past 6 months, children who were coming in level IV and V according to Gross Motor Function Classification System and those who had uncontrolled seizures since past six months. Based on the inclusion criteria, 40 subjects were selected as study participants, and after the selection of participants, they were randomly allocated to group A and group B. The flow chart of the study procedure is mentioned in Figure 1. 

Outcome measures

The outcome measures were recorded on subjects in both the groups at two different instants (pre- and post-treatment) using the Paediatric Balance Scale (PBS), Palpation Meter device (PALM), and Gait Parameters by the assessor who was blinded to the treatment intervention.

Paediatric Balance Scale (PBS)

It is a scale with 14 items through which the static and dynamic balance of the subjects can be assessed. It is similar to the Berg Balance Scale, but the sequencing of the items is different in both scales. PBS is used in school-going children with an age group of 5-15 years [16].

Palpation Meter Device (PALM)

A palpation meter is an instrument that is used to quantify the extent of disturbance in the geometrical alignment of the pelvis. It is a reliable and valid tool to measure pelvic asymmetry in subjects with numerous musculoskeletal defacements [17].

Gait Parameters

Gait parameters included in the present or current research were cadence (number of steps covered in a minute), stride length (distance between the heel strike of one lower limb and again the same lower limb), and gait velocity (velocity with which an individual is walking) [18].

Intervention

**Figure 2:**
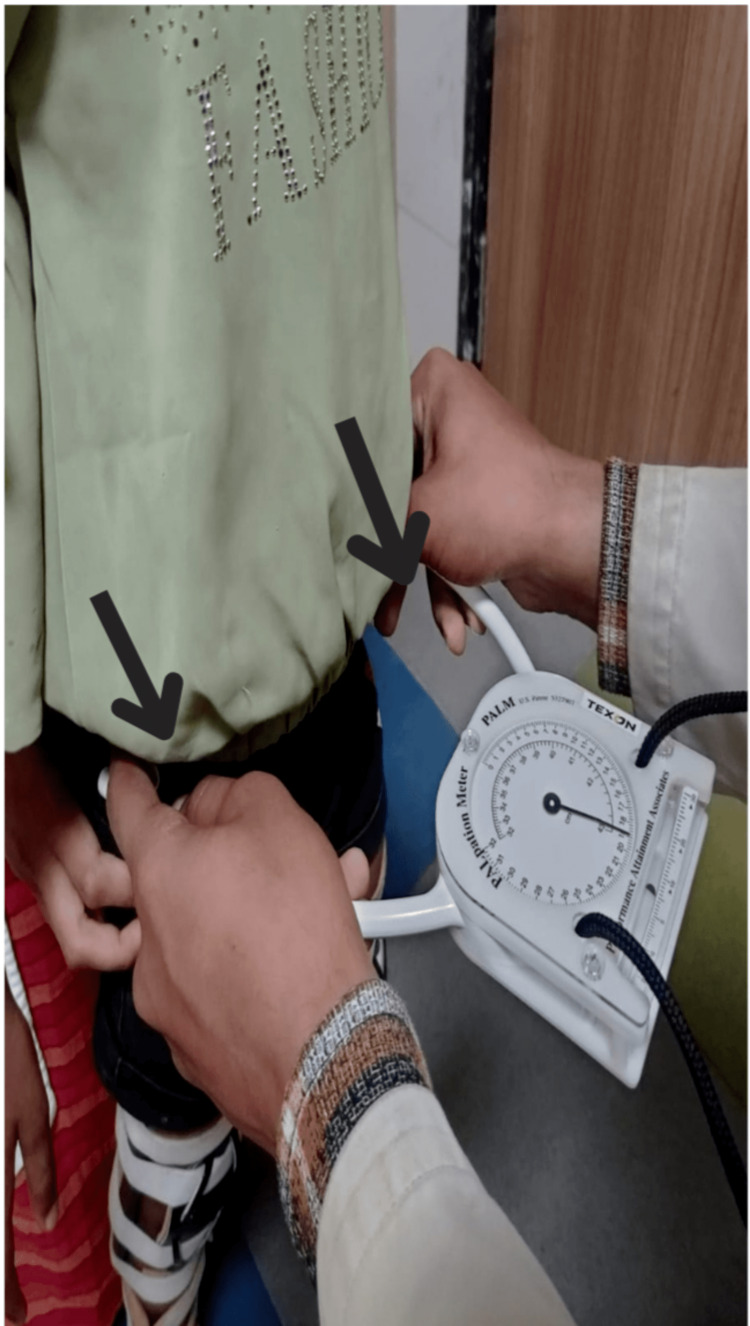
Measurement of pelvic asymmetry with the help of a palpation meter device

Assessment of the subjects was taken at two instants using the same outcome measures (Figure 2), and the subjects allocated in group A were given Pelvic PNF techniques for 15 minutes on both sides six days a week along with Task-Oriented training for 30 minutes, six days a week and continued for four weeks while on the other hand the subjects in group B were given only Task-Oriented training for 30 minutes, six days a week and continued for four weeks.

**Figure 3:**
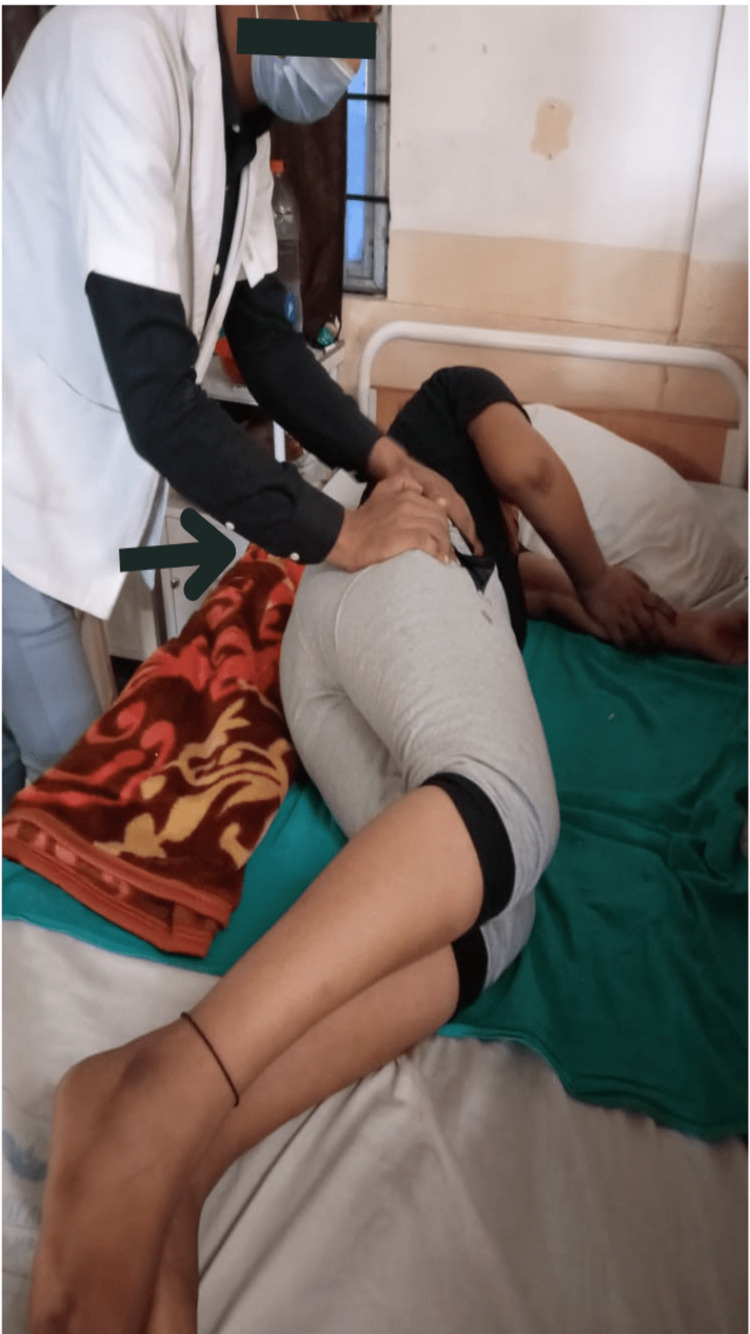
Delivering the techniques of pelvic proprioceptive neuromuscular facilitation in the side-lying position

**Figure 4:**
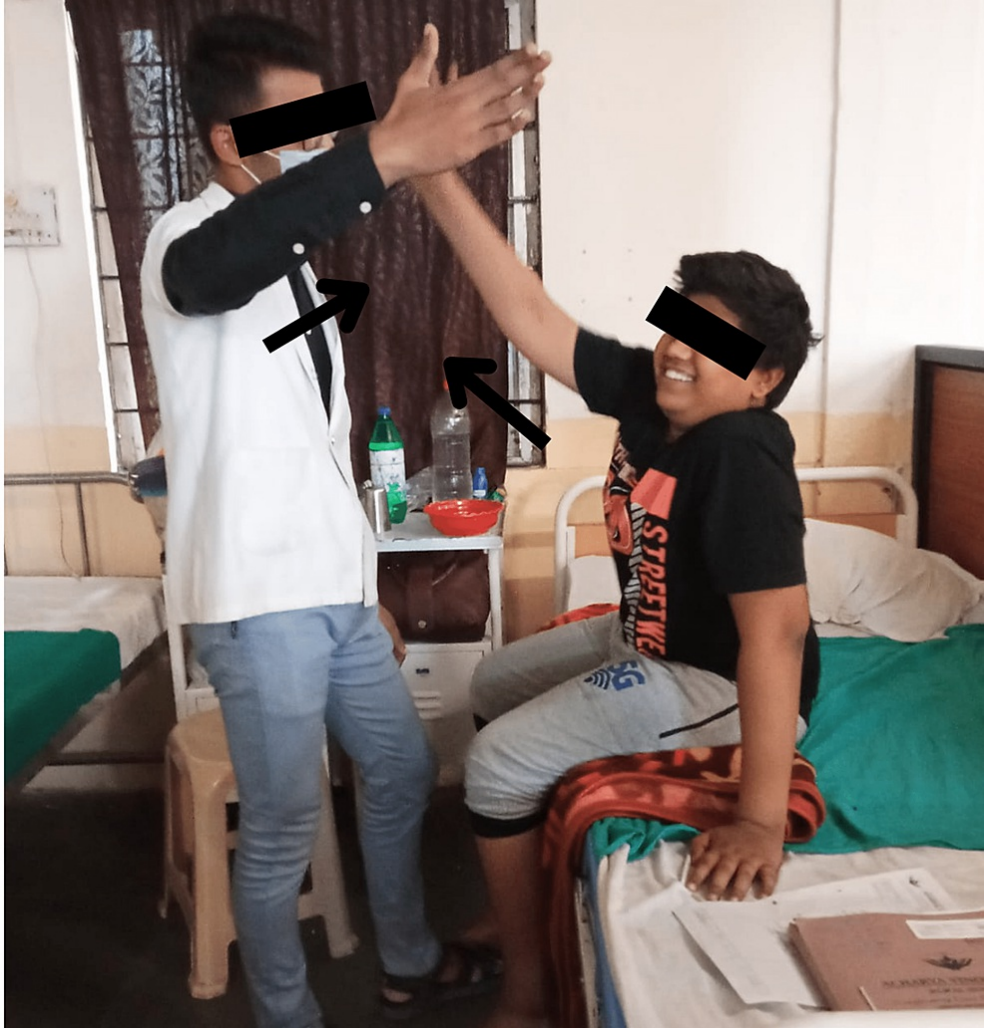
Delivering task-oriented exercises (reaching activities) to the child in a bedside sitting position

Subjects in group A were given Pelvic PNF techniques which included Rhythmic Initiation in which we commenced with passive motion followed by active assisted, active, and then actively resisted movements along with Slow Reversals in which we provided dynamic contractions of the antagonist followed by dynamic contractions of the agonist muscle groups. The techniques of Pelvic PNF were delivered for 15 minutes on both sides six days a week and continued for four weeks (Figures 3, 4). Pelvic PNF techniques were blended with Task-Oriented training, which included sit-to-stand activities, reach-out activities, and walking on the ground. Task-Oriented training or Task-Oriented exercises were given for 30 minutes six days a week and continued for four weeks.

On the other hand, the participants in group B were given only Task-Oriented training, which included sit-to-stand exercises, reach-out activities, and walking on the ground. Task-Oriented training or exercises were given for 30 minutes, six days a week and continuously for four weeks.

## Results

The results were calculated for both groups using a variety of statistical tests and software methods. Statistical tests used to extract the findings of the study were the Chi-Square test and paired and unpaired t-test and the software used for statistical inference was IBM Corp. Released 2020. IBM SPSS Statistics for Windows, Version 27.0. Armonk, NY: IBM Corp and GraphPad Prism 7.0 version, and a p>0.05 were considered. Table 1 shows the division per age group.

**Table 1:** Display of participants in both the groups according to their respective age groups through which it can be inferred that in group A the majority of subjects are of 9 years and in group B the majority of subjects are of 10 years

Age Group(yrs)	Group A	Group B	ϗ2-value
8 yrs	4(20%)	3(15%)	4.21 P=0.31,NS
9 yrs	6(30%)	5(25%)	
10 yrs	5(25%)	8(40%)	
11 yrs	5(25%)	2(10%)	
12 yrs	0(0%)	2(10%)	
Total	20(100%)	20(100%)	
Mean±SD	9.55±1.09	9.75±1.16	
Range	8-11 yrs	8-12 yrs	

Table 2: Division of subjects according to their genders which imply that group A had a majority of male subjects while in group B the proportion of both males and females was uniform (P= 0.33).

**Table 2:** Depiction of participants in both the groups according to their genders through which an inference is made that group A has more number of male subjects while in group B the number of male and female participants is uniform.

Gender	Group A	Group B	ϗ2-value
Male	13(65%)	10(50%)	0.92 P=0.33,NS
Female	7(35%)	10(50%)	
Total	20(100%)	20(100%)	

Table 3: Contrast of baseline and post-treatment readings on the Paediatric Balance Scale from groups A and B, which depicts a marked improvement in the readings of group A (P= 0.001). 

**Table 3:** Display of pre- and post-treatment observations recorded on the Pediatric Balance Scale in both the groups, which is showing a marked upliftment in the values of group A

Group	Before t/t paediatric balance scale	Post t/t paediatric balance scale	Mean Difference	t-value
Group A	45.80±1.15	51.55±1.09	5.75±0.96	26.60 P=0.0001,S
Group B	46.90±1.29	50.40±1.23	3.50±0.60	25.78 P=0.0001,S

Table 4: Contrast of baseline and post-treatment values of cadence recorded in both the groups, which displays a marked upliftment in the values of group A (P= 0.0001).

**Table 4:** Display of pre- and post-intervention cadence values in both the group, which is showing a remarkable improvement in the cadence values of group A

Group	Before t/t cadence	Post t/t cadence	Mean Difference	t-value
Group A	15.20±1.39	27.85±0.74	12.65±1.26	44.61 P=0.0001,S
Group B	15.05±1.14	20±1.65	4.95±1.05	21.08 P=0.0001,S

Table 5: Distinction between the baseline and post-treatment.

**Table 5:** Depiction of pre- and post-treatment gait velocities of both the subjects, which is showing a marked improvement in the gait velocity of group A

Group	Before t/t gait velocity	Post t/t gait velocity	Mean Difference	t-value
Group A	0.41±0.09	1.14±0.06	0.73±0.09	33.35 P=0.0001,S
Group B	0.42±0.08	0.77±0.07	0.35±0.09	15.89 P=0.0001,S

Table 6: Contrast of baseline and post-treatment observations recorded through the Palpation Meter device in which more upliftment was observed in the findings of group A (P= 0.0001).

**Table 6:** Display of PALM readings recorded and documented at pre- and post-treatment instants which is showing a marked enhancement in the values of group A.

Group	Before t/t PALM readings	Post t/t PALM readings	Mean Difference	t-value
Group A	0.67±0.09	0.28±0.06	0.38±0.10	16.55 P=0.0001,S
Group B	0.64±0.12	0.40±0.10	0.24±0.08	13.27 P=0.0001,S

## Discussion

This research has extracted the proof or evidence that the implementation of manoeuvres of Pelvic Proprioceptive Neuromuscular Facilitation gains or achieves a satisfactory impact on the functional potencies in children suffering from Spastic Diplegia. As these children manifest themselves with numerous musculoskeletal issues, such as malalignment of the pelvis, which ultimately hampers and hinders the functional capabilities of these children, such as the potential to withstand or sustain the state of equilibrium and propulsion of body segments in a coordinated pattern in an anterior direction by altering the Base of Support and Centre of Gravity of the body at regular instants [19]. Spasticity favors the hypertonic muscle groups to be in a particular attitude which can lead to the physiological excursion of muscles and further give rise to secondary musculoskeletal defacements [20]. A physiological pattern of walking demands optimal stability as well as control of the pelvis and pelvic manoeuvres of Pelvic PNF in optimizing the Balance and Framework of Gait as well as in rectification of pelvic mal-alignment and restoration of a good pelvic configuration, control, and stability in children suffering from Spastic Diplegic variant of Cerebral Palsy. The results obtained based on statistical analysis had shown a significant difference between the PBS and GP of the interventional group A and the comparator group B. Group A emerged with a remarkable and positive upshot in their respective outcomes (PBS, PALM readings, and Gait Parameters). The midpoint baseline observations on PBS were 45.8 for class A and 46.9 for class B. PALM readings were 0.67 inches for class A and 0.64 inches for class B. Cadence was 15.2 steps/ minute. The stride span was 44.2 for class A cm and 43.45 cm for class B. Gait Velocity of 0.41 m/s for class A and 0.42 m/s for group B.

The midpoint of post-intervention observations for Group A was 51.55 on PBS, 0.28 inches on PALM, 1.14 m/s on Gait Velocity, 27.8 steps/ minute on Cadence, and 59.7 cm on stride length. On the other hand, for group B the observations were 50.4 on PBS, 0.40 inches on PALM, 0.77 m/s on Gait Speed, 20 steps/minute on Cadence, and 50.1 cm on Stride Span. The hypothesis emphasizing the potency of Pelvic PNF manoeuvres to uplift pelvic control and stability was made based on a study conducted by Sukumar and Shilna in the year 2020 in which they emphasized optimizing the control of the trunk in children or subjects with Spastic Diplegia.

Kovela et al. conducted a case report in 2020 to draw the effectiveness of PNF techniques on lower extremity functions in a 19-year-old male suffering from Spastic Diplegia, which is appearing to be in line with the current study [21].

Eek et al. did a study in 2008 to draw the efficacy of muscle strength instruction in promoting the walking abilities of 16 children with the Spastic variant of Cerebral Palsy, which seems to be in line with the current study [22].

Sharma et al. conducted a case report on the efficacy of Proprioceptive Neuromuscular Facilitation to improve the coordination of lower limbs seems to be in resemblance to the current study [23].

Eun et al. did a study in the year 2019 to extract the potential of Group Task-Oriented Training to optimize Gross and Fine motor functioning capacities, activities of daily living, and social inclusion, which seems to be in line with the current study [24]. 

Study limitations

The small sample size of the study was a limitation. More sample size can be drawn to investigate the efficacy of pelvic Proprioceptive Neuromuscular Facilitation on Spastic diplegic Cerebral Palsy. As this was a time-bound research, the follow-up of the intervention cannot be drawn.

## Conclusions

This research has provided evidence regarding the fruitfulness of maneuvers of Proprioceptive Neuromuscular Facilitation of the pelvis in optimizing the variables of gait and balance and in the restoration of the anatomical alignment of the pelvis. The study has proved that when task-oriented training is merged with the maneuvers of Pelvic Proprioceptive Neuromuscular Facilitation (PNF), it imposes an add-on effect in uplifting the variables of gait and balance, thereby it assists the child in achieving a normal functional potential so that the child can cope with the other children of his or her age groups in the context of the abilities or potencies to execute various activities like running, jumping and many more. Therefore, from this research, it can be extracted that Pelvic PNF imposes an advantageous outcome in the rectification of pelvic mal-alignment as a result of any underlying pathology, and it can be an appropriate intervention in the rectification and restoration of the mal-aligned pelvis. As a healthy gait requires optimal coordination of lower limbs and synchronization between the pelvis and inferior limbs, and the current research has created evidence that Pelvic PNF improves the synchrony between the pelvis and inferior limbs.

## References

[1] Sadowska M, Sarecka-Hujar B, Kopyta I (2020). Cerebral palsy: Current opinions on definition, epidemiology, risk factors, classification and treatment options. Neuropsychiatr Dis Treat.

[2] Patel DR, Neelakantan M, Pandher K, Merrick J (2020). Cerebral palsy in children: a clinical overview. Transl Pediatr.

[3] Hallman-Cooper JL, Rocha Cabrero F (2022). Cerebral palsy. https://pubmed.ncbi.nlm.nih.gov/30844174/.

[4] Stavsky M, Mor O, Mastrolia SA, Greenbaum S, Than NG, Erez O (2017). Cerebral palsy-trends in epidemiology and recent development in prenatal mechanisms of disease, treatment, and prevention. Front Pediatr.

[5] Chauhan A, Singh M, Jaiswal N, Agarwal A, Sahu JK, Singh M (2019). Prevalence of cerebral palsy in Indian children: A systematic review and meta-analysis. Indian J Pediatr.

[6] de Morais Filho MC, Kawamura CM, Andrade PH, Dos Santos MB, Pickel MR, Neto RB (2009). Factors associated with pelvic asymmetry in transverse plane during gait in patients with cerebral palsy. J Pediatr Orthop B.

[7] Bhimani R, Anderson L (2014). Clinical understanding of spasticity: implications for practice. Rehabil Res Pract.

[8] Park EY (2020). Stability of the gross motor function classification system in children with cerebral palsy for two years. BMC Neurol.

[9] Eliasson AC, Krumlinde-Sundholm L, Rösblad B, Beckung E, Arner M, Ohrvall AM, Rosenbaum P (2006). The Manual Ability Classification System (MACS) for children with cerebral palsy: scale development and evidence of validity and reliability. Dev Med Child Neurol.

[10] Tschirren L, Bauer S, Hanser C, Marsico P, Sellers D, van Hedel HJ (2018). The Eating and Drinking Ability Classification System: concurrent validity and reliability in children with cerebral palsy. Dev Med Child Neurol.

[11] Bottos M, Feliciangeli A, Sciuto L, Gericke C, Vianello A (2001). Functional status of adults with cerebral palsy and implications for treatment of children. Dev Med Child Neurol.

[12] Crosbie J, Vachalathiti R (1997). Synchrony of pelvic and hip joint motion during walking. Gait Posture.

[13] Hägglund G, Hollung SJ, Ahonen M (2022). Treatment of spasticity in children and adolescents with cerebral palsy in Northern Europe: a CP-North registry study. BMC Neurol.

[14] Anttila H, Autti-Rämö I, Suoranta J, Mäkelä M, Malmivaara A (2008). Effectiveness of physical therapy interventions for children with cerebral palsy: a systematic review. BMC Pediatr.

[15] A N, Shanmugam S, P SR (2020). Effectiveness of pelvic proprioceptive neuromuscular facilitation on trunk control in children with spastic diplegia: A randomized controlled trial. Indian J Public Health Res Dev.

[16] Yi SH, Hwang JH, Kim SJ, Kwon JY (2012). Validity of pediatric balance scales in children with spastic cerebral palsy. Neuropediatrics.

[17] Petrone MR, Guinn J, Reddin A, Sutlive TG, Flynn TW, Garber MP (2003). The accuracy of the Palpation Meter (PALM) for measuring pelvic crest height difference and leg length discrepancy. J Orthop Sports Phys Ther.

[18] Klejman S, Andrysek J, Dupuis A, Wright V (2010). Test-retest reliability of discrete gait parameters in children with cerebral palsy. Arch Phys Med Rehabil.

[19] Graham HK, Thomason P, Willoughby K (2021). Musculoskeletal pathology in cerebral palsy: A classification system and reliability study. Children (Basel).

[20] Gajdosik CG, Cicirello N (2001). Secondary conditions of the musculoskeletal system in adolescents and adults with cerebral palsy. Phys Occup Ther Pediatr.

[21] Krishna KR, Thakur A, Priya PR, Hs S, Srivastav S, Prabhu S (2020). Effect of PNF in improving lower extremity function in adolescent with spastic diplegic cerebral palsy-A case report. Indian J Public Health Res Dev.

[22] Eek MN, Tranberg R, Zügner R, Alkema K, Beckung E (2008). Muscle strength training to improve gait function in children with cerebral palsy. Dev Med Child Neurol.

[23] Sharma P (2021). PNF training for improving lower limb coordination in cerebral palsy: A case study in a child with spastic diplegia. Int J Sci Healthc Res.

[24] Ko EJ, Sung IY, Moon HJ, Yuk JS, Kim HS, Lee NH (2020). Effect of group-task-oriented training on gross and fine motor function, and activities of daily living in children with spastic cerebral palsy. Phys Occup Ther Pediatr.

